# Three‐Year Long‐Term Outcomes in Patients With Unresectable Hepatocellular Carcinoma Treated With Atezolizumab Plus Bevacizumab Treatment in Clinical Practice

**DOI:** 10.1002/cam4.71640

**Published:** 2026-02-15

**Authors:** Hideko Ohama, Atsushi Hiraoka, Toshifumi Tada, Masashi Hirooka, Kazuya Kariyama, Joji Tani, Masanori Atsukawa, Koichi Takaguchi, Ei Itobayashi, Shinya Fukunishi, Kunihiko Tsuji, Toru Ishikawa, Kazuto Tajiri, Hironori Tanaka, Hidenori Toyoda, Chikara Ogawa, Takashi Nishimura, Takeshi Hatanaka, Satoru Kakizaki, Kazuhito Kawata, Atsushi Naganuma, Hisashi Kosaka, Tomomitsu Matono, Hidekatsu Kuroda, Yutaka Yata, Hiroki Nishikawa, Michitaka Imai, Tomoko Aoki, Hironori Ochi, Hideyuki Tamai, Shohei Komatsu, Fujimasa Tada, Shinichiro Nakamura, Yoshiko Nakamura, Teruki Miyake, Osamu Yoshida, Kazuhiro Nouso, Asahiro Morishita, Norio Itokawa, Tomomi Okubo, Taeang Arai, Akemi Tsutsui, Takuya Nagano, Kazunari Tanaka, Takanori Matsuura, Yuichi Koshiyama, Yuki Kanayama, Hidenao Noritake, Hirayuki Enomoto, Kosuke Matsui, Masaki Kaibori, Takumi Fukumoto, Yoichi Hiasa, Masatoshi Kudo, Takashi Kumada, the RELPEC Group and HCC 48 Group

**Affiliations:** ^1^ Department of Gastroenterology Ehime Prefectural Central Hospital Matsuyama Japan; ^2^ Department of Internal Medicine Japanese Red Cross Himeji Hospital Himeji Japan; ^3^ Division of Gastroenterology, Department of Internal Medicine Kobe University Graduate School of Medicine Kobe Japan; ^4^ Department of Gastroenterology and Metabology Ehime University Graduate School of Medicine Toon Japan; ^5^ Department of Gastroenterology Okayama City Hospital Okayama Japan; ^6^ Department of Gastroenterology and Neurology Kagawa University Kagawa Japan; ^7^ Division of Gastroenterology and Hepatology, Department of Internal Medicine Nippon Medical School Tokyo Japan; ^8^ Department of Hepatology Kagawa Prefectural Central Hospital Takamatsu Japan; ^9^ Department of Gastroenterology Asahi General Hospital Asahi Japan; ^10^ Department of Gastroenterology Hyogo Medical University Nishinomiya Japan; ^11^ Center for Gastroenterology Teine Keijinkai Hospital Sapporo Japan; ^12^ Department of Gastroenterology Saiseikai Niigata Hospital Niigata Japan; ^13^ Department of Gastroenterology Toyama University Hospital Toyama Japan; ^14^ Department of Gastroenterology Takarazuka City Hospital Takarazuka Hyogo Japan; ^15^ Department of Gastroenterology and Hepatology Ogaki Municipal Hospital Ogaki Japan; ^16^ Department of Gastroenterology Japanese Red Cross Takamatsu Hospital Takamatsu Japan; ^17^ Department of Gastroenterology Gunma Saiseikai Maebashi Hospital Maebashi Japan; ^18^ Department of Clinical Research NHO Takasaki General Medical Center Takasaki Japan; ^19^ Hepatology Division, Department of Internal Medicine II Hamamatsu University School of Medicine Hamamatsu Japan; ^20^ Department of Gastroenterology NHO Takasaki General Medical Center Takasaki Japan; ^21^ Department of Hepatobiliary Surgery Kansai Medical University Hirakata Japan; ^22^ Department of Hepatology Harima Himeji General Medical Center Himeji Japan; ^23^ Division of Gastroenterology and Hepatology, Department of Internal Medicine Iwate Medical University School of Medicine Morioka Japan; ^24^ Department of Gastroenterology Hanwa Memorial Hospital Osaka Japan; ^25^ Department of Gastroenterology Osaka Medical and Pharmaceutical University Osaka Japan; ^26^ Department of Gastroenterology Niigata Prefectural Cancer Center Niigata Japan; ^27^ Department of Gastroenterology and Hepatology Kindai University Faculty of Medicine Osaka Japan; ^28^ Center for Liver‐Biliary‐Pancreatic Disease Matsuyama Red Cross Hospital Matsuyama Japan; ^29^ Department of Hepatology Wakayama Rosai Hospital Wakayama Japan; ^30^ Division of Hepato‐Biliary‐Pancreatic Surgery, Department of Surgery Kobe University Graduate School of Medicine Kobe Japan; ^31^ Gifu Kyoritsu University Ogaki Japan; ^32^ Department of Epidemiology, Infectious Disease Control, and Prevention Hiroshima University Institute of Biomedical and Health Sciences Hiroshima Japan

**Keywords:** atezolizumab plus bevacizumab, durvalumab plus tremelimumab, hepatocellular carcinoma, IMbrave150 trial, prognosis

## Abstract

**Background:**

Findings regarding long‐term outcomes of unresectable hepatocellular carcinoma (uHCC) patients treated with atezolizumab and bevacizumab (Atez/Bev) have yet to be reported. This study was performed to evaluate results regarding 3 year survival of such patients treated in real‐world clinical settings.

**Methods:**

This multicenter retrospective study included 555 patients with Child‐Pugh A and BCLC stage B or C, for whom Atez/Bev treatment was initiated in the period from 2020 to 2021. Best treatment response, progression‐free survival (PFS), overall survival (OS), post‐progression survival (PPS), and immune‐related adverse events (irAEs) were analyzed.

**Results:**

Median age was 73 years and 80.2% were male. Atez/Bev was given as first‐line therapy in 55.3%. Objective response rate (ORR) was 41.3% and disease control rate (DCR) was 79.4%. Median PFS was 6.2 months, while median OS was 21.6 months with a 3 year survival rate of 29.7%. Patients treated with Atez/Bev as first‐line therapy showed a higher 3 year survival rate of 35.9%. Post‐progression treatment was administered to 54.1% of the patients and median PPS in those was 10.9 months. Conversion therapy was performed in 5.9%. IrAEs occurred in 13.3% (grade 5: 0.7%).

**Conclusions:**

uHCC patients treated with Atez/Bev in clinical practice settings showed good ORR and DCR, as well as favorable long‐term survival with a 3 year survival rate of approximately 30%, including 35.9% for first‐line users.

**Abbreviations**
AEs: Adverse events
Atez/Bev: atezolizumab plus bevacizumab
BCLC: Barcelona Clinic Liver Cancer
CR: complete response
CT: computed tomography
CTCAE: the Common Terminology Criteria for Adverse Events
DCR: disease control rate
Dur: durvalumab
Dur/Tre: durvalumab plus tremelimumab
EOB‐ MRI: gadolinium ethoxybenzyl diethylenetriamine pentaacetic acid‐enhanced magnetic resonance imaging
HAIC: hepatic arterial infusion chemotherapy
HBV: hepatitis B virus
HCC: Hepatocellular carcinoma
HCV: Hepatitis C virus
ICI: immune‐checkpoint inhibitor
IQR: interquartile range
irAEs: immune‐related adverse events
JSH: Japan Society of Hepatology
LEN: lenvatinib
mALBI: modified albumin‐bilirubin
MTA: molecular targeted agent
MWA: microwave ablation
ORR: objective response rate
OS: overall survival
PD: progressive disease
PFS: progression‐free survival
PR: partial response
RECIST: the Response Evaluation Criteria in Solid Tumors
RFA: radiofrequency ablation
SD: stable disease
SOR: sorafenib
TACE: transcatheter arterial chemoembolization
Tre: tremelimumab
uHCC: unresectable HCC

## 1 Introduction

Hepatocellular carcinoma (HCC) is well known as the sixth most common type of cancer, and third or fourth leading cause of cancer death worldwide [1, 2, 3]. Even after undergoing curative treatments (e.g., surgical resection, ablation), recurrence is common, with unresectable HCC (uHCC) status often noted. Recent developments in systemic therapy options that use molecular targeted agent (MTA) and immune‐checkpoint inhibitor (ICI) treatments for uHCC have improved the prognosis of many affected patients. Based on the results of the IMbrave150 trial [4], sorafenib (SOR) and lenvatinib (LEN), given as an initial systemic chemotherapeutic MTA regimen, and atezolizumab plus bevacizumab (Atez/Bev) received approval in 2020 for use as a first regimen including ICI.

More recently, durvalumab (Dur) plus tremelimumab (Tre) (Dur/Tre) was introduced as an initial combination immunotherapy, based on the results of the HIMALAYA trial presented in 2023. In Japan, current clinical guidelines recommend either Atez/Bev or Dur/Tre as first‐line systemic therapies for uHCC [5]. The HIMALAYA trial demonstrated a 3 year overall survival (OS) rate of 30.7% with Dur/Tre [6], providing a benchmark for long‐term outcomes. On the other hand, because the IMbrave150 trial was terminated early due to the obvious superiority of Atez/Bev over sorafenib, long‐term survival data, such as the HIMALAYA trial, have not yet been reported in patients treated with Atez/Bev.

The present study was conducted to evaluate long‐term survival (3 years) of patients who received Atez/Bev therapy in real‐world clinical practice settings.

## 2 Materials and Methods

### 2.1 Study Population

Medical records of 1391 Japanese patients with uHCC who received Atez/Bev at participating centers from September 2020 to December 2024 were examined. Of the 1391 patients, 1372 were enrolled, after excluding data lacking baseline characteristics and prognosis (Figure S1) and the 1372 patients with available clinical data are presented in (Table S1) and Figure S2, respectively. Following exclusion of patients with Child‐Pugh grade B or C and Barcelona Clinic Liver Cancer (BCLC) stage [7] 0, A, or D, and also those who started Atez/Bev therapy after 2022, uHCC patients who received Atez/Bev for more than 3 years were enrolled for analysis of long‐term treatment outcomes. None had a history of ICI treatment. A total of 555 Child‐Pugh class A patients with uHCC (BCLC stage B or C) who received Atez/Bev from 2020 to 2021 were included in the present analysis. As a sub‐analysis, the prognosis of HCC patients with Child‐Pugh class B (*n* = 72), who were treated during the same period and had the same BCLC stage, was evaluated.

When findings showed positive for the presence of the hepatitis B virus (HBV) surface antigen, the etiology of HCC was determined as due to HBV infection. Patients with an HBV infection were typically treated with a nucleotide analog, such as entecavir or tenofovir alafenamide fumarate. Hepatitis C virus (HCV) infection was defined by the presence of anti‐HCV antibodies, and such affected patients were categorized as HCC due to HCV. For patients without a viral infection but with a history of alcohol abuse (≥ 60 g/day for males, ≥ 40 g/day for females) [8], the etiology was attributed to alcohol‐related HCC. Cases for which no specific etiological factor was identified were classified as “others”.

### 2.2 Assessment of Liver Function

Liver function was assessed using the Child‐Pugh classification, and patients with grade A were selected. To further refine the evaluation of liver function, modified albumin‐bilirubin (mALBI) grade was utilized, which subdivides the ALBI grade 2 category into subgrades 2a and 2b based on an ALBI score cut‐off of −2.27 [9].

### 2.3 Diagnosis and Staging for HCC

HCC was diagnosed based on guidelines established by the Japan Society of Hepatology (JSH) [5] and confirmed by use of combinations of clinical, radiological, and/or pathological findings. Radiological diagnosis was determined based on characteristic enhancement patterns observed in findings obtained with dynamic computed tomography (CT), gadolinium ethoxybenzyl diethylenetriamine pentaacetic acid‐enhanced magnetic resonance imaging (EOB‐ MRI) [10], or contrast‐enhanced ultrasonography with perflubutane [11]. Histological confirmation was performed when necessary, using pathological specimens obtained during the clinical course. Tumor staging was determined according to the BCLC system [7].

### 2.4 Treatment Protocol

Before starting Atez/Bev therapy, patients were examined for autoimmune disorders and esophagogastric varices (EGV) to minimize immune‐related adverse events (irAEs) and gastrointestinal hemorrhage occurrence. Any high‐risk EGV identified was managed in a prophylactic manner. Atezolizumab (1200 mg) and bevacizumab (15 mg/kg) were administered intravenously every 3 weeks, in accordance with the IMbrave150 trial protocol and the manufacturer's recommendation.

Adverse events (AEs) were monitored and classified using the Common Terminology Criteria for Adverse Events (CTCAE), version 5.0. For patients with severe or intolerable AEs, the implicated agent was suspended while assessing causality, with treatment discontinued when unacceptable toxicity or disease progression was observed. Tumor response was evaluated using the Response Evaluation Criteria in Solid Tumors (RECIST), version 1.1. Decisions regarding subsequent management following discontinuation of Atez/Bev were left to the attending physician's discretion.

### 2.5 Ethical Approval and Informed Consent

The present study was conducted in accordance with the Declaration of Helsinki and received approval from the institutional review board of each participating center. Written informed consent was obtained following approval from the clinical research committee before beginning treatment.

### 2.6 Statistical Analysis

All analytical data were obtained from a medical records database. Continuous variables are expressed as the median with interquartile range (IQR). Statistical analyses were performed depending on the context, with a Kruskal‐Wallis test used for continuous variables, a chi‐square test combined with Fisher's exact test used for categorical variables, and the Kaplan–Meier method or a log‐rank test used for survival analysis. Prognostic outcomes were assessed based on progression‐free survival (PFS), defined as the time from start of Atez/Bev therapy to diagnosis of disease progression, OS, time from initiation of Atez/Bev treatment to death, and post‐progression survival (PPS), time from diagnosis of disease progression during Atez/Bev treatment to death. Landmark analyses were applied to mitigate immortal time bias. To evaluate the association between OS and PPS, Spearman's rank correlation coefficient (rho) was calculated.

All statistical analyses were performed using EZR, version 1.61 (Saitama Medical Center, Jichi Medical University, Saitama, Japan) [12]. EZR provides a graphical user interface for R (The R Foundation for Statistical Computing, Vienna, Austria) and is a customized version of R Commander, enhanced with additional biostatistical tools.

## 3 Results

### 3.1 Patient Characteristics

The median observation period was 17.8 months (IQR 10.5–31.8). The patients included in this study had a median age of 73 years (IQR 68–79) and included 445 males (80.2%). The etiology of liver disease was viral in 288 (51.9%) and non‐viral in 267 (48.1%). Liver function, assessed using mALBI grade, was grade 1 in 202 (36.4%), grade 2a in 165 (29.7%), grade 2b in 188 (33.9%), and grade 3 in 0 (0%). Regarding HCC stage, 207 patients (37.3%) were classified as BCLC stage B and 348 (62.7%) as stage C. A total of 307 patients (55.3%) received Atez/Bev as first‐line treatment (Table 1).

**TABLE 1:** Clinical characteristics of enrolled patients with Child‐Pugh class A and BCLC‐B/C.

	All (*n* = 555)	First‐line treatment (*n* = 307)
Age, years^a^	73 (68–79)	73 (68–79)
Male: female	445: 110	237: 70
ECOG PS, 0: 1: 2	459: 85: 11	257: 41: 9
Etiology, HBV: HCV: alcohol: other	113: 175: 111: 156	51: 107: 63: 86
Esophageal varices	106, 19.1%	54, 17.6%
ALBI score^a^	−2.43 (−2.70 to −2.17)	−2.48 (−2.73 to −2.23)
mALBI grade, 1: 2a: 2b: 3	202: 165: 188: 0	119: 97: 91
AST, U/L^a^	38 (27–54)	38 (26–58)
ALT, U/L^a^	27 (18–43)	28 (19–44)
Platelets, 10^4^/μL^a^	14.2 (10.9–19.3)	13.9 (10.6–19.3)
Total bilirubin, mg/dL^a^	0.8 (0.6–1.0)	1.0 (0.6–1.0)
Albumin, g/dL^a^	3.8 (3.4–4.1)	4.0 (3.5–4.1)
Prothrombin time^a^	91.6 (82.7–100.0)	88.0 (80.0–97.6)
FIB‐4 index^a^	3.71 (2.57–5.35)	3.79 (2.59–5.72)
NLR^a^	2.68 (1.80–3.72)	2.52 (1.74–3.68)
AFP, ng/mL^a^	53.0 (6.0–768.85)	26.0 (5.6–510.7)
DCP, mAU/mL^a^	392.0 (54.5–3626.0)	235.0 (37.0–3659.5)
MVI	124, 22.3%	75, 24.4%
EHM	230, 41.4%	113, 36.8%
BCLC stage, B: C	207:348	127:180
Treatment line, first: later	307:248	307:0

Abbreviations: AFP, alpha‐fetoprotein; ALBI, albumin‐bilirubin; ALT, alanine aminotransferase; AST, aspartate aminotransferase; BCLC, Barcelona Clinic Liver Cancer; DCP, des‐gamma‐carboxy prothrombin; ECOG PS, Eastern Cooperative Oncology Group performance status; EHM, extra‐hepatic metastasis; HBV, hepatitis B virus; HCV, hepatitis C virus; mALBI, modified albumin‐bilirubin; MVI, major vessel invasion; NLR, neutrophil‐to‐lymphocyte ratio.

^a^ Median (interquartile range).

### 3.2 Therapeutic Best Response

Best treatment response was evaluated in 499 patients, with complete response (CR), partial response (PR), stable disease (SD), and progressive disease (PD) noted in 31, 175, 190, and 103, respectively. The objective response rate (ORR) was 41.3% and disease control rate (DCR) was 79.4%.

### 3.3 Safety of Atez/Bev Treatment

Greater than 10% of the patients were affected by AEs, including proteinuria, fatigue, hypertension, appetite loss, liver dysfunction, rash, edema/ascites, and fever (Table 2). AEs classified as Grade 3 or higher were observed in 182 (32.8%). Furthermore, irAEs were observed in 74 patients (13.3%), with incidence details presented in (Table 3). The median time to onset of irAE was 3.03 months (IQR 0.93–6.23). Grade 5 irAEs were observed in four patients (0.7%), including interstitial pneumonia in two, polymyositis in one, and bullous pemphigoid in one. The median time to onset of Grade 5 irAEs was 2.28 months (IQR: 0.80–6.46). None of the four patients had a history of autoimmune disease, and all had a baseline liver function of Child‐Pugh score 5. One case of polymyositis occurred during first‐line Atez/Bev treatment, while the other three cases received Atez/Bev as second‐line therapy. The best treatment response was SD in three cases and PD in one case. High dose steroids were used for irAE treatment in 17 (3.1% of entire cohort) of these patients.

**TABLE 2:** Adverse events.

	All grades	≥ Grade 3
Proteinuria	218 (39.3%)	69 (12.4%)
Fatigue	142 (25.6%)	15 (2.7%)
Hypertension	114 (20.5%)	24 (4.3%)
Appetite loss	107 (19.3%)	13 (2.3%)
Liver disfunction	92 (16.6%)	27 (4.8%)
Rash	71 (12.8%)	8 (1.4%)
Edema/ascites	70 (12.6%)	20 (3.6%)
Fever	63 (11.4%)	6 (1.1%)

**TABLE 3:** Immune‐related adverse events.

	All grades	≥ Grade 3
Adrenal hypofunction	12 (2.2%)	4 (0.7%)
Rash/skin disorder	9 (1.6%)	5 (0.9%) (grade 5, *n* = 1)
Interstitial pneumonia	9 (1.6%)	6 (1.1%) (grade 5, *n* = 2)
Liver disfunction	9 (1.6%)	4 (0.7%)
Colitis/diarrhea	6 (1.1%)	2 (0.4%)
Renal failure	4 (0.7%)	2 (0.4%)
Thyroid dysfunction	4 (0.7%)	0 (0.0%)
Myositis/rhabdomyolysis	3 (0.5%)	2 (0.4%) (grade 5, *n* = 1)
Myocarditis/cardiac failure	3 (0.5%)	1 (0.2%)
Fever	2 (0.4%)	1 (0.2%)
Organ hemorrhage	2 (0.4%)	0 (0.0%)
Arthritis	2 (0.4%)	0 (0.0%)
Ascites	1 (0.2%)	1 (0.2%)
Hypopituitarism	1 (0.2%)	0 (0.0%)
Pancreatitis	1 (0.2%)	1 (0.2%)
Type 1 diabetes	1 (0.2%)	0 (0.0%)
Pharyngitis	1 (0.2%)	1 (0.2%)
Neuritis	1 (0.2%)	0 (0.0%)
Cheilitis	1 (0.2%)	0 (0.0%)
Lower limb weakness	1 (0.2%)	0 (0.0%)
Appetite loss	1 (0.2%)	0 (0.0%)

*Note:* Among the 555 enrolled patients, four (0.7%) had a grade 5 immune‐related adverse event (irAE), including skin disorder in one, interstitial pneumonia in two, and myositis/rhabdomyolysis in one.

### 3.4 PFS and OS

**FIGURE 1:**
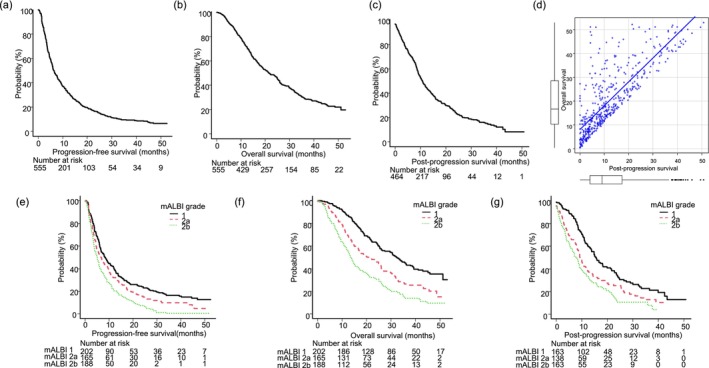
Three‐year survival outcomes for all patients with unresectable hepatocellular carcinoma who received atezolizumab plus bevacizumab therapy (*n* = 555). (a) Median progression‐free survival was 6.2 months (95% CI: 5.6–7.1). (b) Median overall survival was 21.6 months (95% CI: 19.0–25.0), with a 3 year survival rate of 29.7% (95% CI: 25.5–34.0). (c) Median post‐progression survival was 10.9 months (95% CI: 9.6–12.3). (d) A strong positive correlation was observed between post‐progression survival and overall survival (Spearman's rho = 0.808, *p* < 0.001). (e) Median progression‐free survival for mALBI grade 1, 2a, and 2b was 8.1 months (95% CI: 6.5–10.6), 6.5 months (95% CI: 4.7–8.3), and 5.0 months (95% CI: 3.9–5.9), respectively (*p* < 0.01). (f) Median overall survival for mALBI grade 1, 2a, and 2b was 32.8 months (95% CI: 26.1–38.8), 20.1 months (95% CI: 16.2–25.9), and 13.6 months (95% CI: 12.0–16.1), respectively (*p* < 0.01). (g) Median post‐progression survival for mALBI grade 1, 2a, and 2b was 15.7 months (95% CI: 13.5–18.9), 9.4 months (95% CI: 8.2–11.5), and 8.1 months (95% CI: 6.0–9.0), respectively (*p* < 0.01).

Median PFS for all 555 patients was 6.2 months (95% CI: 5.6–7.1) and median OS was 21.6 months (95% CI: 19.0–25.0), with a 3 year survival rate of 29.7% (95% CI: 25.5–34.0) (Figure 1a,b).

When PFS and OS were stratified based on mALBI grade, significant differences in prognosis were observed. Median PFS for mALBI grade 1 was 8.1 months (95% CI: 6.5–10.6), for grade 2 was 6.5 months (95% CI: 4.7–8.3), and for grade 2b was 5.0 months (95% CI: 3.9–5.9) (*p* < 0.001), while similarly, median OS for grade 1 was 32.8 months (95% CI: 26.1–38.8), for grade 2a was 20.1 months (95% CI: 16.2–25.9), and for grade 2b was 13.6 months (95% CI: 12.0–16.1) (*p* < 0.001) (Figure 1e,f). As well as in the patients with CP‐B, PFS was 5.0 months (95% CI: 3.6–7.0) and median OS was 9.8 months (95% CI: 7.3–12.8), with a 3 year survival rate of 13.2% (95 CI: 6.1%–23.2%) (Figure S3).

### 3.5 Post‐Progression Treatment and Survival

Based on RECIST criteria, 464 patients were classified as PD during the clinical course. Of those, post‐progression treatment was administered to 300 (54.1%), including other systemic therapies in 190 (40.9%), surgery in five (1.1%), radiofrequency ablation (RFA) or microwave ablation (MWA) in 10 (2.2%), transcatheter arterial chemoembolization (TACE) or hepatic arterial infusion chemotherapy (HAIC) in 61 (13.1%), beyond PD treatments in 23 (5.0%), and radiotherapy in 11 (2.3%). Median PPS for these 464 patients was 10.9 months (95% CI: 9.6–12.3) (Figure 1c). A strong positive correlation was observed between PPS and OS (Spearman's rho = 0.808, *p* < 0.001), indicating that post‐progression survival was a major contributor to overall survival in this cohort (Figure 1d).

In the patients with CP‐B, median PPS was 5.5 months (95% CI: 3.7–8.6), and significant correlation with OS was observed (Spearman's rho = 0.736, *p* < 0.001) (Figure 1g).

Additionally, median PPS showed significant differences according to mALBI grade, as that for grade 1 was 15.7 months (95% CI: 13.5–18.9), for grade 2a was 9.4 months (95% CI: 8.2–11.5), and for grade 2b was 8.1 months (95% CI: 6.0–9.1) (*p* < 0.001). Furthermore, the rate of transition to post‐progression treatment was 76.1% for mALBI grade 1, 64.5% for grade 2a, and 53.4% for grade 2b (*p* < 0.001), again significantly different among the groups (Figure 1g).

### 3.6 Conversion Therapy

Conversion therapy was performed in 24 patients (4.3%), including surgery in nine and RFA or MWA in 15.

### 3.7 OS Based on Treatment Line, Presence or Absence of irAEs, and Best Treatment Response

Median OS for patients who received the Atez/Bev therapy as first‐line treatment was 25.1 months (95% CI: 20.5–30.3), with a 3 year survival rate of 35.9% (95% CI: 29.8–42.0). In contrast, median OS for patients who received the Atez/Bev therapy as a later line was 18.7 months (95% CI: 16.5–23.6), with a 3 year survival rate of 22.8% (95% CI: 17.4–28.7), indicating a significant difference between first‐ and later‐line therapy (*p* = 0.007) (Figure S4).

Regarding the effects of irAEs, median OS was 20.9 months (95% CI: 17.8–24.5) in patients without irAEs, with a 3 year survival rate of 29.2% (95% CI: 24.7–33.8), while median OS was 26.5 months (95% CI: 19.9–33.2) and the 3 year survival rate was 32.8% (95% CI: 21.7–44.3) in those with irAEs (*p* = 0.711). In addition, when patients with irAEs were divided into three groups—those without irAEs, those with Grade 1–2 irAEs, and those with Grade ≥ 3 irAEs—the median OS was 20.9 months (95% CI: 17.8–24.5), 25.4 months (95% CI: 17.8–32.2), and 32.2 months (95% CI: 23.8–39.3), respectively. No significant differences were observed among the three groups (*p* = 0.924). Furthermore, no significant differences in OS were observed when patients were categorized by the type of irAE (colitis, liver dysfunction, dermatologic disorders, interstitial pneumonia, endocrine disorders, and others) (*p* = 0.251).

**FIGURE 2:**
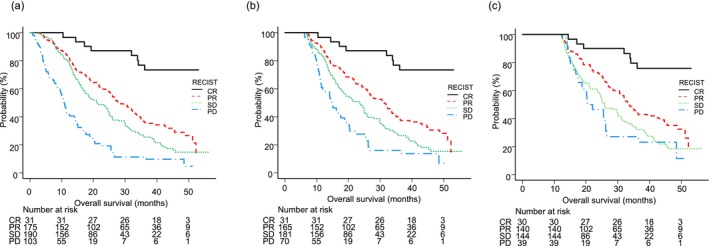
Overall survival (OS) according to best treatment response according to Response Evaluation Criteria in Solid Tumors (RECIST). (a) Median OS for the complete response (CR), partial response (PR), stable disease (SD), and progressive disease (PD) groups was NA, 28.5 months, 21.1 months, and 10.8 months, respectively, showing significant differences among them (*p* < 0.001). (b) Median OS in 6 month landmark for the CR, PR, SD, and PD groups was NA, 30.6 months, 23.6 months, and 14.8 months, respectively, showing significant differences among them (*p* < 0.001). (c) Median OS at the 12 month landmark for the CR, PR, SD, and PD groups was NA, 33.6 months, 25.9 months, and 22.1 months, respectively, showing significant differences among them (*p* < 0.001).

OS was also evaluated based on best treatment response according to RECIST. In the CR group, median OS was not achieved (NA) (95% CI: NA‐NA) and the 3 year survival rate was 77.0% (95% CI: 57.8–88.3), while that in the PR group was 28.5 months (95% CI: 24.1–33.4) and the 3 year survival rate was 36.1% (95% CI: 28.2–43.9); in the SD group was 21.1 months (95% CI: 17.3–25.0) and the 3 year survival rate was 25.5% (95% CI: 18.6–32.9); and in the PD group was 10.8 months (95% CI: 8.6–14.0) and the 3 year survival rate was 11.3% (95% CI: 5.3–19.8). Significant differences were observed among the groups (*p* < 0.001) (Figure 2a). In 6 month landmark analysis, median OS for the CR, PR, SD, and PD groups was NA (95% CI: NA‐NA), 30.6 months (95% CI: 26.0–34.8), 23.6 months (95% CI: 18.4–25.1), and 14.8 months (95% CI: 11.5–18.7), respectively, indicating a significant difference among them (*p* < 0.001) (Figure 2b). Similarly, 12 month landmark analysis results showed that median OS for the CR, PR, SD, and PD groups was NA (95% CI: NA‐NA), 33.6 months (95% CI: 30.5–42.5), 25.9 months (95% CI: 23.9–31.0), and 22.1 months (95% CI: 17.6–26.1), respectively, again with a significant difference among them observed (*p* < 0.001) (Figure 2c).

### 3.8 Treatment Outcomes for First‐Line Treatment

The baseline characteristics of the 307 patients who received Atez/Bev as first‐line treatment are presented in (Table 1). Treatment response evaluations showed that 19 achieved CR and 108 achieved PR, while there were 93 with SD and 53 with PD. ORR was 41.4% and DCR was 71.7%. IrAEs were observed in 42 patients (13.7%), of whom 8 required high dose steroid treatment.

**FIGURE 3:**
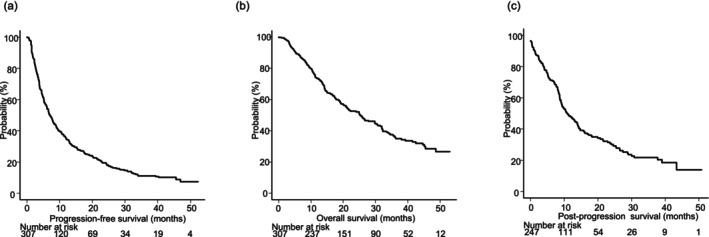
Three‐year survival outcomes for patients with unresectable hepatocellular carcinoma who received atezolizumab plus bevacizumab as first‐line treatment. (a) Median progression‐free survival was 6.8 months (95% CI: 5.8–8.1). (b) Median overall survival was 25.1 months (95% CI: 20.5–30.3), with a 3 year survival rate of 35.9% (95% CI: 29.8–42.0). (c) Median post‐progression survival was 11.0 months (95% CI: 9.2–13.9).

Median PFS was 6.8 months (95% CI: 5.8–8.1) and median OS was 25.1 months (95% CI: 20.5–30.3) with a 3 year survival rate of 35.9% (95% CI: 29.8–42.0). Transition to post‐progression treatment was noted in 66.4% (164 of 247 patients with PD noted in RECIST) and median PPS for those was 11.0 months (95% CI: 9.2–13.9) (Figure 3).

**FIGURE 4:**
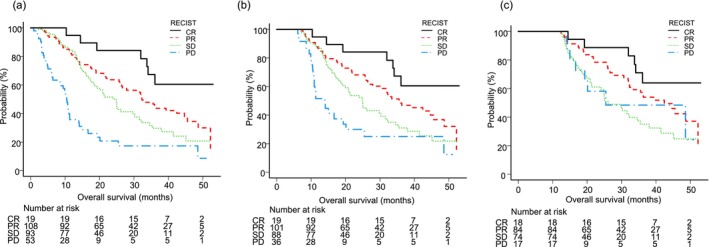
Overall survival (OS) according to best treatment response according to Response Evaluation Criteria in Solid Tumors (RECIST) in patients with unresectable hepatocellular carcinoma who received atezolizumab plus bevacizumab as first‐line treatment. (a) Median OS for the complete response (CR), partial response (PR), stable disease (SD), and progressive disease (PD) groups was NA, 32.3 months, 24.9 months, and 10.5 months, respectively, showing significant differences among them (*p* < 0.001). (b) Median OS at the 6 month landmark for the CR, PR, SD, and PD groups was NA, 35.6 months, 24.9 months, and 14.1 months, respectively, showing significant differences among them (*p* < 0.001). (c) Median OS at the 12 month landmark for the CR, PR, SD, and PD groups was NA, 42.5 months, 26.1 months, and 25.4 months, respectively, showing significant differences among them (*p* = 0.008).

Finally, OS was evaluated based on best treatment response according to RECIST. Median OS was NA (95% CI: 33.8‐NA) for the CR group, 32.3 months (95% CI: 26.0–44.7) for the PR group, 24.9 months (95% CI: 18.5–30.3) for the SD group, and 10.5 months (95% CI: 6.4–11.4) for the PD group, showing significant differences among them (*p* < 0.001). Six‐ and 12 month landmark analyses showed that median OS for each group remained consistent, again with significant differences among them (respectively, *p* < 0.001 and *p* = 0,008) (Figure 4).

## 4 Discussion

The present study showed a favorable outcome for long‐term (3 year) OS in uHCC patients treated with Atez/Bev. Overall, the 3 year survival rate for patients with uHCC treated with Atez/Bev was 29.7%, and 35.9% among those who received it as first‐line therapy. Although this was a retrospective study conducted in a real‐world setting and not a head‐to‐head comparison, the 3 year survival outcome observed in the present cohort may be comparable to that reported for Dur/Tre in the HIMALAYA trial (30.7%) [6]. In the HCC treatment guidelines proposed by the Japan Society of Hepatology (JSH), both Atez/Bev and Dur/Tre are recommended as first‐line treatments for uHCC, as no randomized controlled trials have directly compared these two regimens. To the best of our knowledge, this is the first study to report long‐term (3 year) outcomes of uHCC patients treated with Atez/Bev in clinical practice.

Additionally, the present results showed ORR of 41.3%, DCR of 79.4%, median PFS of 6.2 months, and incidence rate of grade 3 or higher AEs of 34.2%. These outcomes are comparable to previously reported data in terms of both therapeutic efficacy and safety [4, 13]. Moreover, median PPS was 10.9 months. Importantly, we found that patients with a better mALBI grade showed a significantly higher post‐progression treatment rate (grade 1, 2a, 2b = 76.1%, 64.5%, 53.4%, respectively, *p* < 0.001) and longer PPS (grade 1, 2a, 2b = median 15.7, 9.4, 8.1 months, respectively, *p* < 0.001). Furthermore, OS was significantly better in patients with better treatment response (CR = NA; PR, SD, PD = 28.5, 21.1, 10.8 months, respectively, *p* < 0.001). These data reinforce the clinical relevance of introducing Atez/Bev therapy while liver function is preserved, particularly in patients with mALBI grade 1 or 2a, to maximize the opportunity for sequential therapy. Prior to approval of Atez/Bev treatment, sorafenib [14] and lenvatinib [15] were developed as initial systemic MTA regimens for uHCC, with regorafenib [16], ramucirumab [17], and cabozantinib [18] developed as later‐line MTA treatment options. In addition, Dur/Tre was approved as a new initial systemic immune treatment without an anti‐VEGF drug. Studies conducted in the recent decade related to uHCC have noted that introduction of systemic treatment for patients with better hepatic function increases the possibility of post‐progression treatment, while sequential treatment following tumor progression for prolonging post‐progression survival is also a very important factor to improve prognosis, not only in the MTA era but also in the immune‐therapy era [19, 20, 21, 22, 23, 24]. Favorable treatment outcomes have been found for newly developed Atez/bev [25] and Dur/Tre [26] in clinical trials, with additional post‐progression treatments performed after treatment failure in several patients noted in each of those reports. In the present study, a significant strong correalation between PPS and OS was observed in CP‐A patients (Spearman's rho = 0.808, *p* < 0.001), while in CP‐B patients, PPS was shorter compared to those with CP‐A. Furthermore, Tajiri K et al. [27] showed that PPS was a more important factor (R2 = 0.872, *p* < 0.001) than objective response or PFS (R2 = 0.605, *p* = 0.001) in the reported clinical traials with immune therapies, with the same noted in the MTA era [20], indicating that the clinical significance of post‐progression treatment has remained important regardless of immune‐therapy era.

In the clinical practice findings, patients administered lenvatinib as second‐line treatment after Atez/Bev showed a good outcome (median OS 15.7 months, 95% CI 9.6‐NA), while there was no significant difference in overall survival as compared to other MTAs (13.6 months, 95% CI 8.5‐NA) (*p* = 0.992) [28]. A study of renal cell carcinoma cases found that median PFS when using cabozantinib post‐treatment after MTA failure was 7.4 months [29], while that after nivolumab failure was 11.5 months [30], indicating better prognosis in the latter cases and that a pseudo‐combination effect is possible. On the other hand, it was shown that use of Dur/Tre as later‐line treatment after Atez/Bev failure might not be a good therapeutic option [31]. A study of ICI rechallenge in hepatocellular carcinoma cases found that the therapeutic effect varied without any particular trend depending on response to previous treatments or regimen used, with ORR and DCR of second‐line ICI 26% and 55%, respectively [32]. On the other hand, a very good prognosis (CR/PR) was noted for 22% of patients who failed ICI treatment and received combined immunotherapy as post‐treatment [33]. At the time of writing, the effectiveness of sequential ICI therapies remains controversial, thus it will be necessary to accumulate additional clinical data to clarify the optimal order of use for MTA and ICI regimens. Nevertheless, the present findings indicate that long‐term survival of uHCC patients treated with Atz/Bev as the initial line is favorable, as the 3 year rate for the present cases was 35.9%.

The present report has some limitations, including being conducted in a retrospective manner. Furthermore, the results were obtained from a hospital‐based population. Also, the multicenter nature of our cohort, where treatments for AEs, choice of post‐progression treatments, and timing of transitions were left to the discretion of the attending physician, is another limitation. Future prospective studies are needed to consider the effects of strategies for Atez/Bev treatment in combination with subsequent treatment.

In conclusion, findings similar to those obtained in the IMbrave150 trial indicating good treatment response and safety were noted for patients who received uHCC treatment with Atez/Bev in real‐world clinical practice. Importantly, our study is the first to demonstrate 3 year survival outcomes including AE information in the real‐world setting and shows that preserved liver function at baseline enhances access to post‐progression therapies and prolongs PPS. Additional research is needed to identify clinical features of patients who are most likely to benefit from Atez/Bev treatment, as well as for the establishment of appropriate strategies for the use of Atez/Bev and Dur/Tre.

## Author Contributions

**Hideko Ohama:** conceptualization (equal), data curation (equal), formal analysis (equal), investigation (equal), writing – original draft (equal), writing – review and editing (equal). **Atsushi Hiraoka:** data curation (equal), formal analysis (equal), supervision (equal), writing – review and editing (equal). **Toshifumi Tada:** data curation (equal). **Masashi Hirooka:** data curation (equal). **Kazuya Kariyama:** data curation (equal). **Joji Tani:** data curation (equal). **Masanori Atsukawa:** data curation (equal). **Koichi Takaguchi:** data curation (equal). **Ei Itobayashi:** data curation (equal). **Shinya Fukunishi:** data curation (equal). **Kunihiko Tsuji:** data curation (equal). **Toru Ishikawa:** data curation (equal). **Kazuto Tajiri:** data curation (equal). **Hironori Tanaka:** data curation (equal). **Hidenori Toyoda:** data curation (equal). **Chikara Ogawa:** data curation (equal). **Takashi Nishimura:** data curation (equal). **Takeshi Hatanaka:** data curation (equal). **Satoru Kakizaki:** data curation (equal). **Kazuhito Kawata:** data curation (equal). **Atsushi Naganuma:** data curation (equal). **Hisashi Kosaka:** data curation (equal). **Tomomitsu Matono:** data curation (equal). **Hidekatsu Kuroda:** data curation (equal). **Yutaka Yata:** data curation (equal). **Hiroki Nishikawa:** data curation (equal). **Michitaka Imai:** data curation (equal). **Tomoko Aoki:** data curation (equal). **Hironori Ochi:** data curation (equal). **Hideyuki Tamai:** data curation (equal). **Shohei Komatsu:** data curation (equal). **Fujimasa Tada:** data curation (equal). **Shinichiro Nakamura:** data curation (equal). **Yoshiko Nakamura:** data curation (equal). **Teruki Miyake:** data curation (equal). **Osamu Yoshida:** data curation (equal). **Kazuhiro Nouso:** data curation (equal). **Asahiro Morishita:** data curation (equal). **Norio Itokawa:** data curation (equal). **Tomomi Okubo:** data curation (equal). **Taeang Arai:** data curation (equal). **Akemi Tsutsui:** data curation (equal). **Takuya Nagano:** data curation (equal). **Kazunari Tanaka:** data curation (equal). **Takanori Matsuura:** data curation (equal). **Yuichi Koshiyama:** data curation (equal). **Yuki Kanayama:** data curation (equal). **Hidenao Noritake:** data curation (equal). **Hirayuki Enomoto:** data curation (equal). **Kosuke Matsui:** data curation (equal). **Masaki Kaibori:** data curation (equal). **Takumi Fukumoto:** data curation (equal). **Yoichi Hiasa:** data curation (equal). **Masatoshi Kudo:** data curation (equal). **Takashi Kumada:** data curation (equal), supervision (equal).

## Funding

The authors have nothing to report.

## Disclosure

Assistant Editor of Hepatology Research: Toshifumi Tada is an editorial board member of Hepatology Research.

## Ethics Statement

The consent procedure used in the present study was reviewed and approved by the Institutional Ethics Committee of NHO Takasaki General Medical Center (approval number: TGMC2024‐00, date of decision: 2024/04/24). After receiving official approval, this study was conducted as a retrospective analysis of database records based on the Guidelines for Clinical Research issued by the Ministry of Health and Welfare of Japan. All procedures were done in accordance with the declaration of Helsinki. The data were made anonymous before analysis to protect patient privacy.

## Consent

Prior to obtaining approval from the clinical research committee, informed consent was obtained from each participant using an opt‐out method and written consent was then secured after receiving approval. Data were anonymized before analysis to safeguard patient confidentiality.

## Conflicts of Interest

Atsushi Hiraoka, MD, PhD: lecture fees; Eli Lilly, AstraZeneca, and Chugai. Toshifumi Tada, MD, PhD: lecture fees; AbbVie, Eisai, AstraZeneca, and Chugai. Masanori Atsukawa, MD, PhD: lecture fees; AbbVie, Gilead, and Aska Pharmaceutical. Satoru Kakizaki, MD, PhD: lecture fees; AbbVie. Hidenori Toyoda, MD, PhD: lecture fees; Eisai, Chugai, Takeda, Terumo, AbbVie, Gilead, Fujifilm WAKO, Kowa, Bayer, and AstraZeneca. Masatoshi Kudo, MD, PhD: honoraria; Chugai, Eisai, Eli Lilly, AstraZeneca, and Takeda; and research funding; AbbVie, Eisai, GE Healthcare, Otsuka, Taiho, and Chugai; and consulting fees; Chugai, Roche, Eisai, and AstraZeneca. None of the other authors have potential conflicts of interest to declare. Assistant Editor of Hepatology Research. Toshifumi Tada is an editorial board member of Hepatology Research.

## Supporting information


**Figure S1:** Flowchart of patient selection process. Atez/Bev: atezolizumab plus bevacizumab, HCC: hepatocellular carcinoma, BCLC: Barcelona Clinic Liver Cancer.
**Figure S2:** Progression‐free and overall survival for all patients with unresectable hepatocellular carcinoma who received atezolizumab plus bevacizumab at participating centers from September 2020 to December 2024. mPFS: median progression‐free survival, mOS: median overall survival, 95% CI: 95% confidence interval. (a) Progression‐free survival. (b) Overall survival. (c) Progression‐free survival according to mALBI grade. (d) Overall survival according to mALBI grade.
**Figure S3:** Progression‐free, overall and post‐progression survival for Child‐Pugh B patients with unresectable hepatocellular carcinoma who received atezolizumab plus bevacizumab at participating centers from September 2020 to December 2021. mPFS: median progression‐free survival, mOS: median overall survival, mPPS: median post‐progression survival, 95% CI: 95% confidence interval. (a) Progression‐free survival. (b) Overall survival. (c) Post‐progression survival.
**Figure S4:** Overall survival (OS) according to treatment line in unresectable hepatocellular carcinoma patients treated with atezolizumab plus bevacizumab. Median OS was 25.1 months in the first‐line group, significantly longer than in the late‐line group at 18.7 months (*p* = 0.007).

**Table S1:** Clinical characteristics of patients with unresectable hepatocellular carcinoma treated with atezolizumab plus bevacizumab (*n* = 1372).

## Data Availability

Due to the nature of the research, the datasets generated and/or analyzed are not publicly available. Since participants could not be contacted regarding sharing of findings publicly, supporting data, including datasets generated and/or analyzed, are not publicly accessible. However, these datasets can be obtained from the corresponding author upon reasonable request.
