# Supplementary material for: Three‐Year Long‐Term Outcomes in Patients With Unresectable Hepatocellular Carcinoma Treated With Atezolizumab Plus Bevacizumab Treatment in Clinical Practice
Source: Cancer Med. 2026 Feb 15;15(2):e71640. doi: 10.1002/cam4.71640 (PMC12906954; doi:10.1002/cam4.71640)
Supplement: Supplementary file 1 — Figure S1: Flowchart of patient selection process. Atez/Bev: atezolizumab plus bevacizumab, HCC: hepatocellular carcinoma, BCLC: Barcelona Clinic Liver Cancer. Figure S2: Progression‐free and overall survival for all patients with unresectable hepatocellular carcinoma who received atezolizumab plus bevacizumab at participating centers from September 2020 to December 2024. mPFS: median progression‐free survival, mOS: median overall survival, 95% CI: 95% confidence interval. (a) Progression‐free survival. (b) Overall survival. (c) Progression‐free survival according to mALBI grade. (d) Overall survival according to mALBI grade. Figure S3: Progression‐free, overall and post‐progression survival for Child‐Pugh B patients with unresectable hepatocellular carcinoma who received atezolizumab plus bevacizumab at participating centers from September 2020 to December 2021. mPFS: median progression‐free survival, mOS: median overall survival, mPPS: median post‐progression survival, 95% CI: 95% confidence interval. (a) Progression‐free survival. (b) Overall survival. (c) Post‐progression survival. Figure S4: Overall survival (OS) according to treatment line in unresectable hepatocellular carcinoma patients treated with atezolizumab plus bevacizumab. Median OS was 25.1 months in the first‐line group, significantly longer than in the late‐line group at 18.7 months (p = 0.007). [file CAM4-15-e71640-s002.pptx]

## Slide 1
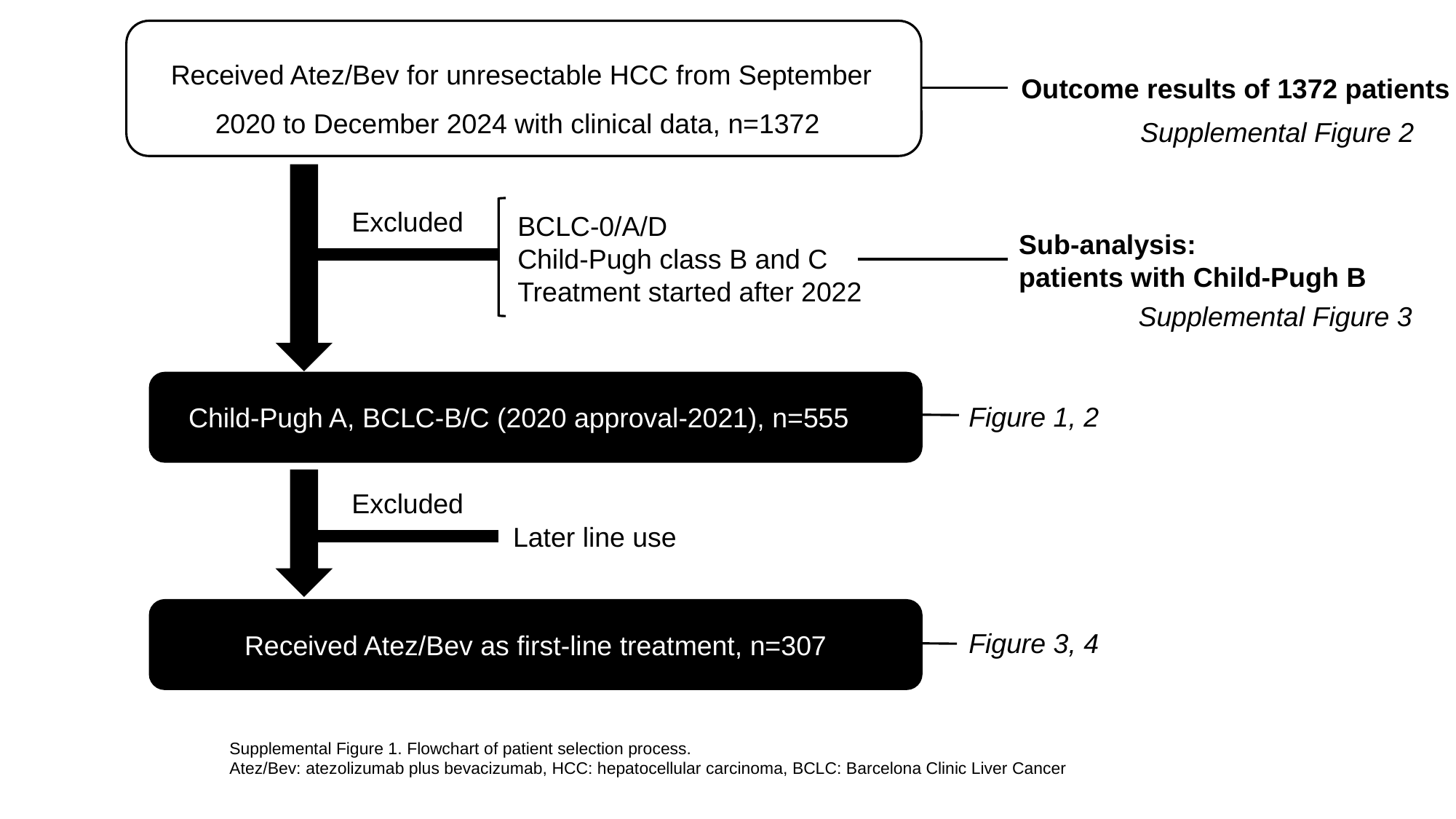

Received Atez/Bev for unresectable HCC from September 2020 to December 2024 with clinical data, n=1372
Outcome results of 1372 patients
Supplemental Figure 2
Excluded
BCLC-0/A/D
Child-Pugh class B and C
Treatment started after 2022
Sub-analysis:
patients with Child-Pugh B
Supplemental Figure 3
Child-Pugh A, BCLC-B/C (2020 approval-2021), n=555
Figure 1, 2
Excluded
Later line use
Received Atez/Bev as first-line treatment, n=307
Figure 3, 4
Supplemental Figure 1. Flowchart of patient selection process.
Atez/Bev: atezolizumab plus bevacizumab, HCC: hepatocellular carcinoma, BCLC: Barcelona Clinic Liver Cancer

## Slide 2
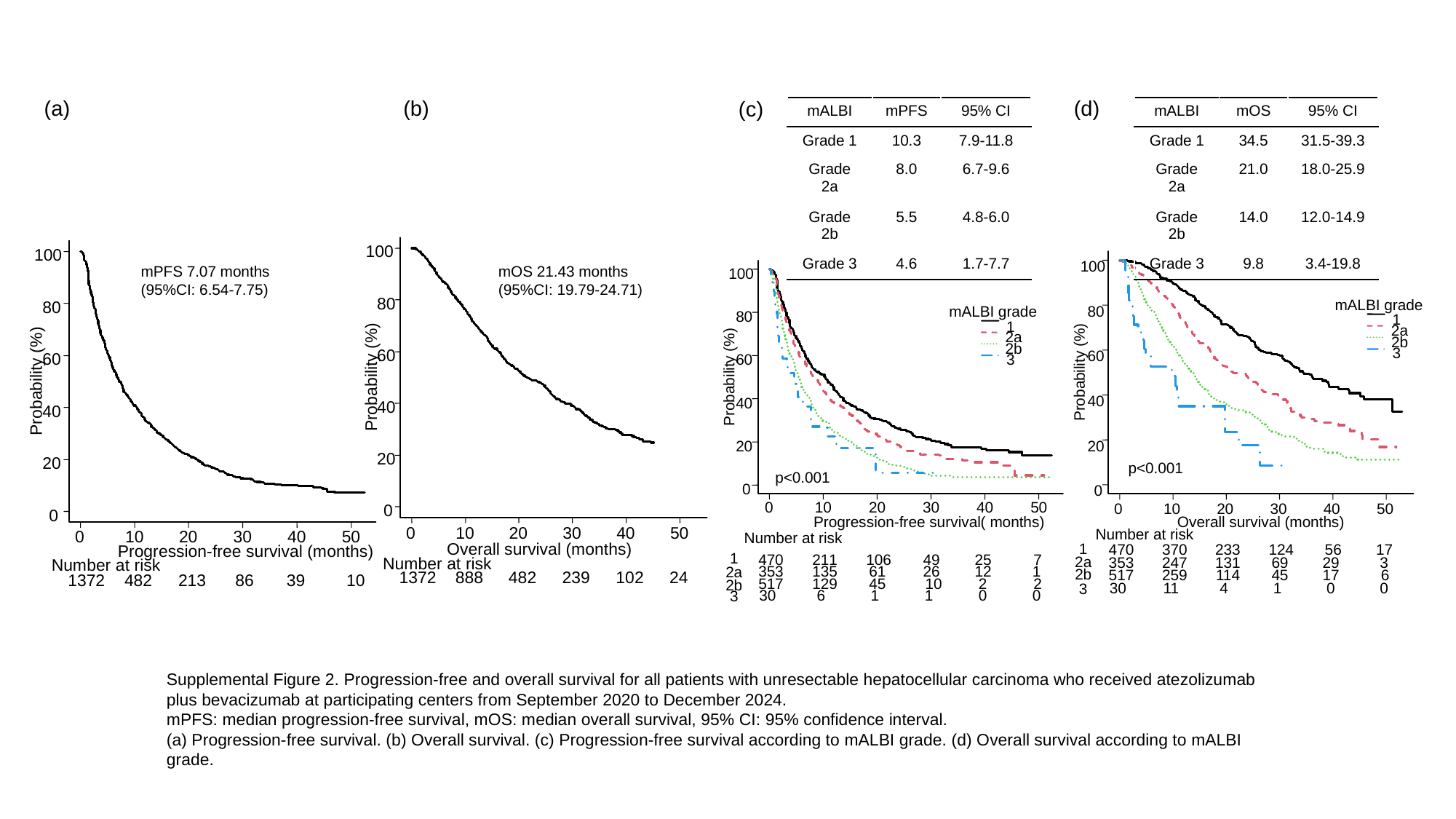

(a)
(b)
(d)
(c)
| mALBI | mPFS | 95% CI |
| --- | --- | --- |
| Grade 1 | 10.3 | 7.9-11.8 |
| Grade 2a | 8.0 | 6.7-9.6 |
| Grade 2b | 5.5 | 4.8-6.0 |
| Grade 3 | 4.6 | 1.7-7.7 |
| mALBI | mOS | 95% CI |
| --- | --- | --- |
| Grade 1 | 34.5 | 31.5-39.3 |
| Grade 2a | 21.0 | 18.0-25.9 |
| Grade 2b | 14.0 | 12.0-14.9 |
| Grade 3 | 9.8 | 3.4-19.8 |
100
80
60
Probability (%)
40
20
0
0
10
20
30
40
50
Overall survival (months)
Number at risk
1372
482
239
102
24
888
100
80
60
Probability (%)
40
20
0
0
10
20
30
40
50
Progression-free survival (months)
Number at risk
482
1372
213
86
39
10
mPFS 7.07 months
(95%CI: 6.54-7.75)
mOS 21.43 months
(95%CI: 19.79-24.71)
100
100
mALBI grade
1
2a
2b
3
80
60
Probability (%)
40
20
p<0.001
0
0
10
20
30
40
50
Progression-free survival( months)
Number at risk
1
470
211
106
49
25
7
353
135
61
26
12
1
2a
517
129
45
10
2
2
2b
30
6
1
1
0
0
3
mALBI grade
1
2a
2b
3
80
60
Probability (%)
40
20
p<0.001
0
0
10
20
30
40
50
Overall survival (months)
Number at risk
1
470
370
233
124
56
17
2a
353
247
131
69
29
3
2b
517
259
114
45
17
6
30
11
4
1
0
0
3
Supplemental Figure 2. Progression-free and overall survival for all patients with unresectable hepatocellular carcinoma who received atezolizumab plus bevacizumab at participating centers from September 2020 to December 2024.
mPFS: median progression-free survival, mOS: median overall survival, 95% CI: 95% confidence interval.
(a) Progression-free survival. (b) Overall survival. (c) Progression-free survival according to mALBI grade. (d) Overall survival according to mALBI grade.

## Slide 3
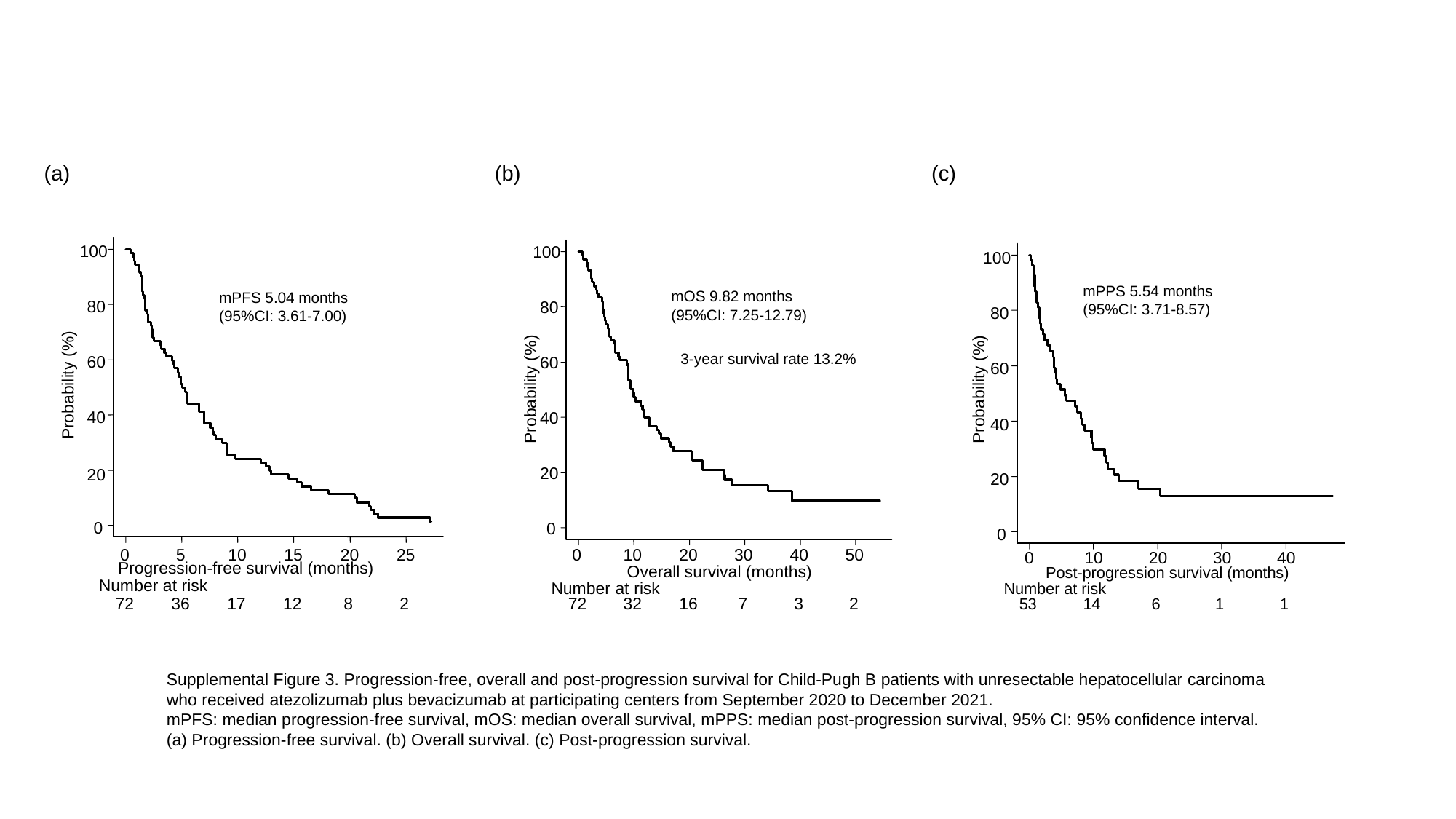

(a)
(b)
(c)
100
80
60
Probability (%)
40
20
0
0
5
10
15
20
25
Progression-free survival (months)
Number at risk
72
36
17
12
8
2
mPFS 5.04 months
(95%CI: 3.61-7.00)
100
mOS 9.82 months
(95%CI: 7.25-12.79)
80
60
Probability (%)
40
20
0
0
10
20
30
40
50
Overall survival (months)
Number at risk
72
32
16
7
3
2
100
mPPS 5.54 months
(95%CI: 3.71-8.57)
80
60
Probability (%)
40
20
0
0
10
20
30
40
Post-progression survival (months)
Number at risk
53
14
6
1
1
3-year survival rate 13.2%
Supplemental Figure 3. Progression-free, overall and post-progression survival for Child-Pugh B patients with unresectable hepatocellular carcinoma who received atezolizumab plus bevacizumab at participating centers from September 2020 to December 2021.
mPFS: median progression-free survival, mOS: median overall survival, mPPS: median post-progression survival, 95% CI: 95% confidence interval.
(a) Progression-free survival. (b) Overall survival. (c) Post-progression survival.

## Slide 4
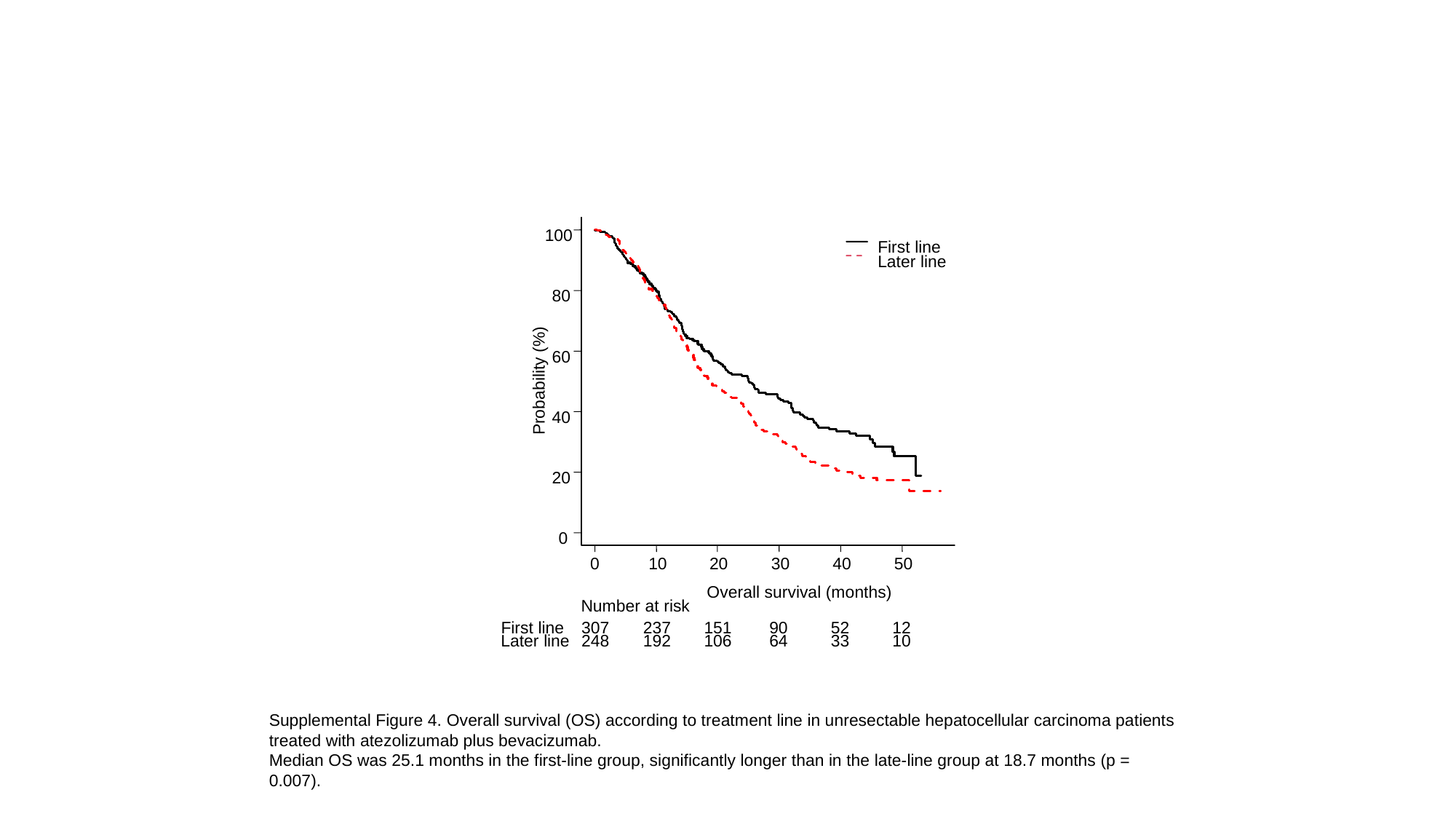

100
First line
Later line
80
60
Probability (%)
40
20
0
0
10
20
30
40
50
Overall survival (months)
Number at risk
First line
307
237
151
90
52
12
Later line
248
192
106
64
33
10
Supplemental Figure 4. Overall survival (OS) according to treatment line in unresectable hepatocellular carcinoma patients treated with atezolizumab plus bevacizumab.
Median OS was 25.1 months in the first-line group, significantly longer than in the late-line group at 18.7 months (p = 0.007).
