# Supplementary material for: Three‐Year Long‐Term Outcomes in Patients With Unresectable Hepatocellular Carcinoma Treated With Atezolizumab Plus Bevacizumab Treatment in Clinical Practice
Source: Cancer Med. 2026 Feb 15;15(2):e71640. doi: 10.1002/cam4.71640 (PMC12906954; doi:10.1002/cam4.71640)
Supplement: Supplementary file 2 — Table S1: Clinical characteristics of patients with unresectable hepatocellular carcinoma treated with atezolizumab plus bevacizumab (n = 1372). [file CAM4-15-e71640-s001.docx]

Supplemental Table 1. Clinical characteristics of patients with unresectable hepatocellular carcinoma treated with atezolizumab plus bevacizumab (n=1372)

| Age, years* | 74 (68-80) |
| --- | --- |
| Male: female | 1086: 286 |
| ECOG PS, 0:1: ≥2 | 1124：211：37 |
| Etiology, HBV:HCV:alcohol:other | 227:451:280:414 |
| Esophageal varices | 217, 15.8% |
| ALBI score* | -2.40 (-2.70 to -2.06) |
| mALBI grade, 1:2a:2b:3 | 470:353:517:30 |
| Child-Pugh class, A: B: C | 1182:186:4 |
| AST, U/L* | 38 (27-57) |
| ALT, U/L* | 28 (18-43) |
| Platelets, 10^4^/µL* | 14.8 (10.9-20.0) |
| Total bilirubin, mg/dL* | 0.8 (0.6-1.1) |
| Albumin, g/dL* | 3.7 (3.3-4.1) |
| Prothrombin time, %* | 90.0 (78.9-100.2) |
| FIB-4 index* | 3.71 (2.55-5.63) |
| NLR* | 2.59 (1.76-3.71) |
| AFP, ng/mL* | 31.1 (5.2-638.1) |
| DCP, mAU/mL* | 291.0 (43.0-3182.0) |
| MVI | 295, 21.5% |
| EHM | 516, 37.6% |
| BCLC stage, 0:A:B:C:D | 19:113:475:746:11 |
| Treatment line, first:later | 956:416 |

*Median (interquartile range). ECOG PS: Eastern Cooperative Oncology Group performance status, HBV: hepatitis B virus, HCV: hepatitis C virus, ALBI: albumin-bilirubin, mALIBI: modified albumin-bilirubin, AST: aspartate aminotransferase, ALT: alanine aminotransferase, NLR: neutrophil-to-lymphocyte ratio, AFP: alpha-fetoprotein, DCP: des-gamma-carboxy prothrombin, MVI: major vessel invasion, EHM: extra-hepatic metastasis, BCLC: Barcelona Clinic Liver Cancer
